# Is a Resorbable Citrate-based Bioceramic Device Associated With Osseous Integration? An Early Retrospective MRI Analysis

**DOI:** 10.1097/CORR.0000000000004016

**Published:** 2026-06-15

**Authors:** Wayne S. Berberian, Jonathan Day, Lew Schon

**Affiliations:** 1 Hackensack Meridian School of Medicine, Nutley, NJ, USA; 2 Mercy Medical Center, Baltimore, MD, USA

## Abstract

**Background:**

Bioresorbable fixation devices may be advantageous because they eliminate the question of whether they ought to be removed, have biomimetic qualities, and minimize artifacts seen on MRI. Most bioresorbable devices utilized during orthopaedic surgery consist of thermoplastic polymers derived from lactic and glycolic acids. These devices are linked to implant-related osteolysis, inflammation, and bone tunnel enlargement, and their integration with surrounding bone is inconsistent. Citregen®, a resorbable composite of a citrate-based thermoset polymer and hydroxyapatite, recently has been approved for use in the United States; yet to date, there are no published studies documenting device-related MRI findings.

**Questions/purposes:**

(1) Was there observable bone tunnel enlargement on MRI at a minimum of 2 years after implantation of a resorbable citrate-based bioceramic device? (2) Were there other observable adverse findings (such as bone marrow edema or device breakage) on MRI at a minimum of 2 years after implantation of a resorbable citrate-based bioceramic device? (3) Did citrate-based bioceramic devices demonstrate integration with surrounding bone on MRI at a minimum of 2 years after implantation? (4) What was the frequency of early clinical complications after soft tissue fixation with citrate-based bioceramic interference devices?

**Methods:**

All 15 patients who underwent tendon transfer or ligament reconstruction in the foot and ankle during a 21-month time period were treated with citrate-based bioceramic interference devices. This group of patients had a median (range) age of 55 years (21 to 75), and 10 were male. Most presented with degenerative or work-related conditions; two had athletic injuries. Two patients were lost to follow-up, and MRI scans performed on 13 patients (25 devices) more than 2 years after surgery were reviewed separately in retrospective, blinded fashion by two investigators. For each device, surrounding bone was assessed for bone tunnel enlargement, which was quantified in millimeters by measuring the width of any gap between the inner surface of the bone tunnel and the outer diameter of the device threads. An intraclass correlation coefficient of 1.0 was achieved, indicating absolute agreement between raters with regard to width of gap measured to the nearest millimeter. The proportion of bone marrow edema abutting the total cross-sectional circumference of each device was quantified using a five-point interval scale (0%, 0% to 25%, 26% to 50%, 51% to 75%, and 76% to 100%). Percentage intervals assessed on the five-point scale demonstrated high interrater reliability (prevalence-adjusted, bias-adjusted kappa [PABAK] = 0.92). Qualitative assessment for other adverse findings, such as cysts, sinus tracts, peridevice fluid, and device breakage, was also performed. The proportion of osseous integration along the long axis of each device was also quantified using a five-point interval scale (0%, 0% to 25%, 26% to 50%, 51% to 75%, and 76% to 100%). Percentage intervals assessed on the five-point scale demonstrated high interrater reliability (PABAK = 0.92). Retrospective chart analysis was used to document the number of complications at the most recent follow-up.

**Results:**

There was no measurable bone tunnel enlargement around any of the devices. Two devices showed bone marrow edema limited to 25% or less of device circumference. Osseous integration occurred around all devices, with 21 of 25 implants showing near-complete (76% to 100%) integration along their long axes. Clinically, there were no device-related complications at median follow-up of 36 months in the 13 patients who were accounted for at a minimum of 2 years (range 2 to 4 years).

**Conclusion:**

In our study of a citrate-based thermoset polymer with hydroxyapatite, common problems associated with other resorbable devices were not seen. Although these findings are promising, our series was very small and had multiple limitations. Therefore, this study should not serve as a basis to recommend widespread use of this material without publication of larger and more rigorous studies that independently replicate our findings.

**Level of Evidence:**

Level IV, therapeutic study.

## Introduction

Bioresorbable devices in orthopaedic surgery offer a variety of theoretical advantages over devices made of metal and nonresorbable polymers such as polyetheretherketone (PEEK). Good bioresorbable fixation devices may be advantageous because they eliminate the question of whether they ought to be removed, more closely approximate mechanical properties of bone, and minimize artifacts seen on MRI. However, thermoplastic polymers derived from lactic and glycolic acids that have been marketed for use in the United States over the past 25 years or more have gained a flawed reputation because of their chemical properties. The lactate-based devices currently used experience bulk degradation in vivo, demonstrating a quality known as “acid dumping.” This poorly controlled process of polymer resorption lowers the pH of surrounding tissue and bone, causing osteolysis [1, 7, 16, 21], inflammation [15, 16, 18], bone tunnel enlargement [3, 12, 19, 24], and device breakage [2]. Furthermore, there is little evidence that these materials support osseous integration [4, 20, 21, 23], and they may actually impede this process (Fig. 1).

**Fig. 1:**
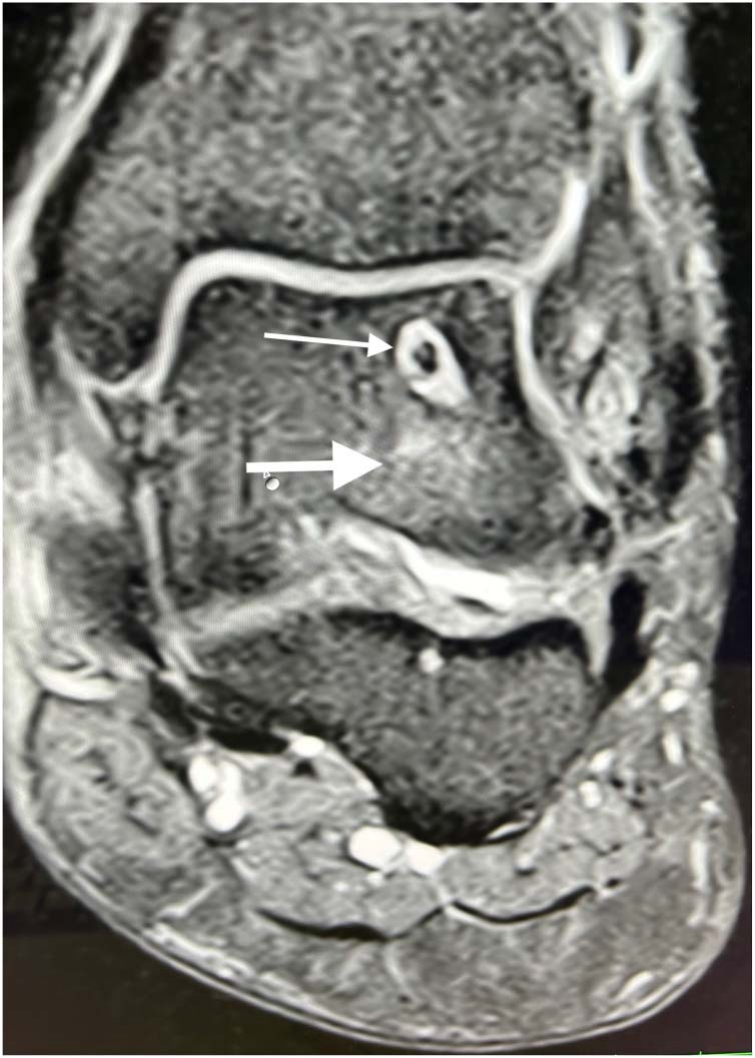
This MRI demonstrates an example of adverse effects of a lactate-based thermoplastic screw 7 years after implantation, with fluid surrounding the device in the talus (thin arrow) and bone marrow edema plantar to the device (thick arrow). The fibular tunnel is also enlarged and fluid filled with no osseous integration.

In October 2020, the FDA granted 510(k) clearance to the first citrate-based bioresorbable orthopaedic fixation device (Fig. 2). Subsequently, the FDA granted clearance to a knotless suture anchor, an ACL interference device, and a bone void filler, all manufactured from the same citrate-based polymer. Unlike all other FDA-approved bioresorbable polymeric devices up to this point, the CitreLock® device (Acuitive Technologies) is not composed of a thermoplastic polymer but is instead comprised of hydroxyapatite fortified with a thermoset polymer called Citregen® (Acuitive Technologies) [17]. In comparison with thermoplastic polymers, thermosets demonstrate a higher compressive strength, experience elastic (reversible) deformation in compression rather than plastic deformation, and exhibit a predictable surface resorption profile [22, 25]. This property prevents acid dumping, and gradually releases calcium, phosphate, and citrate throughout the material’s life cycle [17, 26]. Once implanted, surface erosion of the citrate-based polymer begins immediately through hydrolysis, which gradually progresses inward, leaving behind a bioceramic construct for the body to metabolize as the surrounding bone tunnel remodels [22, 26, 27].

**Fig. 2:**
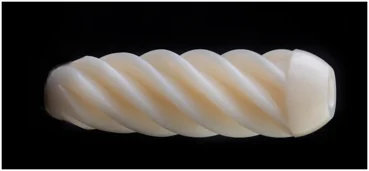
The CitreLock Interference Device (Acuitive Technologies) is composed of a citrate-based bioceramic composite.

Thousands of these citrate-based bioceramic devices have been implanted in patients in the United States over the past 4 years, and yet to date, no peer-reviewed articles have been published on device-related MRI findings. We felt at this early stage of follow-up that it would be important to prioritize MRI over patient-reported outcome measures because entities such as bone marrow edema and osteolysis have been associated with persistent pain and loss of function [8, 10]; and yet, they may be difficult to accurately detect on surveys and physical examination.

In this study, we therefore asked: (1) Was there observable bone tunnel enlargement on MRI at a minimum of 2 years after implantation of a resorbable citrate-based bioceramic device? (2) Were there other observable adverse findings (such as bone marrow edema or device breakage) on MRI at a minimum of 2 years after implantation of a resorbable citrate-based bioceramic device? (3) Did citrate-based bioceramic devices demonstrate integration with surrounding bone on MRI at a minimum of 2 years after implantation? (4) What was the frequency of early clinical complications after soft tissue fixation with citrate-based bioceramic interference devices?

## Patients and Methods

### Study Design and Setting

This was a retrospective case series drawn from the practice of one surgeon (WSB), who was on the design team for the CitreLock Tendon Interference Device (Acuitive Technologies). Patients underwent surgery between September 23, 2021, and June 26, 2023. This surgeon’s practice is approximately 50% administrative and 50% clinical. The clinical portion is dedicated exclusively to orthopaedic foot and ankle surgery but has a wide scope, and only a limited portion involves tendon transfer and augmented ligament reconstruction.

### Patients

A total of 15 patients underwent implantation with at least one citrate-based bioceramic device during the pertinent time period. Of these 15 patients, 13 met all inclusion criteria. Two patients were excluded from the MRI analysis because they did not undergo postoperative MRI scans.

Inclusion criteria consisted of patients who were diagnosed with a tendon or ligament disorder of the ankle or hindfoot that was treated with surgical fixation of a tendon or graft and who underwent that procedure using the device during the above time period. During the study period, the surgeon used only citrate-based bioceramic devices for all ligament reconstructions and tendon transfers in the foot and ankle. No other fixation devices were used by the surgeon for these procedures during that time.

Adjunctive surgical procedures were performed in some patients (as described below), but because the primary objective of this study was to evaluate the effect of the citrate-based bioceramic composite on the bone that surrounds it, the presence of such procedures was not considered a contraindication to study inclusion.

### Descriptive Data

Our cohort of 15 patients (including the two who did not undergo MRI) had a median (range) age of 55 years (21 to 75), and 10 were male (Table 1). Most patients presented with degenerative or work-related conditions, while two had athletic injuries. There were nine patients with Achilles tendinosis, five patients with chronic ligament instability, and one patient who had sustained a deep laceration to his leg 3 months earlier, leading to an equinus contracture and correctable varus deformity. Comorbidities included smoking (n = 4), diabetes (n = 1), morbid obesity (n = 1), cirrhosis (n = 1), preexistent calcaneal osteomyelitis (n = 1), and Ehlers-Danlos syndrome (n = 1).

**Table 1.** Demographic data

Patient	Age in years	Sex	Surgical diagnosis	Comorbidities
1	32	Female	Achilles tendinosis	Ehlers-Danlos syndrome
2	31	Male	Chronic Achilles rupture	Smoking
3	35	Male	Chronic Achilles rupture	
4	55	Male	Lateral ankle ligament instability with varus hindfoot	Diabetes Smoking
5	56	Male	Lateral ankle ligament instability	Obesity
6	44	Female	Lateral ankle ligament instability nonunion of a malleolar fragment	
7	36	Male	Chronic Achilles rupture	History of calcaneal osteomyelitis (treated with multiple surgical procedures) Smoking
8	21	Male	Lateral ankle ligament instability Chronic deltoid ligament rupture Nonunion of the malleolar fragments	
9	69	Female	Achilles tendinosis with Haglund deformity	Hepatic cirrhosis
10	75	Female	Insertional Achilles tendon rupture with severe tendinosis	
11	70	Male	Chronic Achilles rupture	
12	66	Male	Achilles tendon rupture with severe tendinosis	
13	55	Female	Recurrent lateral ligament instability with varus hindfoot Peroneus longus and brevis tendon tears	Retained hardware from previous surgery for lateral ligament repair
14	55	Male	Chronic laceration of peroneus longus and superficial peroneal nerve Varus hindfoot Equinus contracture	
15	36	Male	Proximal Achilles rupture with tendinosis	Smoking

Nine patients underwent surgery for flexor hallucis longus (FHL) tendon transfer, five patients underwent augmented ligament reconstruction with split peroneal tendon autograft, and one patient underwent a split anterior tibialis tendon transfer (SPLATT). Secondary procedures performed concurrently with the tendon transfers or ligament reconstructions included calcaneal osteotomy, excision of bony fragment or Haglund deformity, removal of hardware, peroneal tendon repair or transfer, Achilles tendon lengthening, and excision of superficial peroneal neuroma with placement of nerve conduit (Table 2).

**Table 2.** Surgical procedure and device data

Patient	Surgical procedure(s)	Number of citrate-based bioceramic devices implanted	Total device volume in mm^3^	Total device surface area in mm^2^
1	FHL tendon transfer	1	254	417
2	FHL tendon transfer^a^	1	390	630
3	FHL tendon transfer^a^	1	390	630
4	Augmented lateral ankle ligament reconstruction Calcaneal osteotomy	3	911	1454
5	Augmented lateral ankle ligament reconstruction	3	823	1397
6	Augmented lateral ankle ligament reconstruction Excision of bony fragment	3	686	1183
7	FHL tendon transfer Partial resection of bone from calcaneus	1	390	630
8	Augmented medial and lateral ankle ligament reconstruction Excision of bony fragments	6	1863	2931
9	FHL tendon transfer with Haglund resection	1	390	630
10	FHL tendon transfer with insertional repair of Achilles	1	390	630
11	FHL tendon transfer	1	717	834
12	FHL tendon transfer with repair of Achilles	1	530	724
13	Augmented lateral ligament reconstruction Calcaneal osteotomy Removal of hardware Repair of peroneal tendons	2	295	978
14	Split anterior tibialis tendon transfer Calcaneal osteotomy Achilles tendon lengthening Excision neuroma and superficial peroneal nerve repair with conduit Peroneus longus to brevis tendon transfer	1	161	383
15	FHL tendon transferAchilles repair	1	390	630

a These patients did not undergo postoperative MRI and were not included in the analysis.

### Surgical Technique

One surgeon (WSB) performed all procedures resulting in device implantation. FHL tendon transfer was performed by transecting the tendon proximal to the sustentacular groove, sizing it, and reaming a calcaneal tunnel that was 0.5 mm wider than the tendon. After tensioning the FHL tendon in the tunnel, a citrate-based bioceramic device the same diameter as the tendon was impacted into the tunnel from dorsal to plantar. Fixation for SPLATT was performed in a similar fashion, with the lateral limb of the split anterior tibialis tendon tensioned in a cuboid tunnel that was 0.5 mm wider than the tendon. A citrate-based device the same diameter as the tendon was impacted from dorsal to plantar.

Augmented ligament reconstruction was performed by percutaneous harvest of a split peroneal tendon autograft. The graft was sutured into a triradiate configuration, with the doubled portion fixed in a malleolar tunnel and the free ends fixed in talar and calcaneal tunnels. In all patients, the tunnel was oversized by 0.5 mm, and the device diameter matched that of the tendon graft.

The number of citrate-based bioceramic devices used in each case matched the number of graft fixation sites, and this was determined by the type of surgery that was performed. The size of the devices used varied according to the diameters of the tendon grafts measured during surgery, and thus the diameter of the tunnel that was reamed. The diameters and lengths of the devices used during surgery were recorded for each patient and used to calculate the total implanted volume and surface area of polymer (Table 2).

### MRI Technique

We routinely performed 3T MRI scans of the ankle and hindfoot without gadolinium on all study patients for surveillance purposes at least 2 years after implantation. Mean ± SD time in months between device implantation and MRI scan was 37 ± 6. MRI scans were all performed on the same scanner in the same facility using an identical protocol. This protocol consisted of T1, T2, T2-weighted fat-suppressed, and short tau inversion recovery (STIR) images in the axial, coronal, and sagittal planes. Each device was visualized using T2-weighted fat-suppressed and STIR images.

### Primary and Secondary Study Outcomes

Two principal investigators independently read the scans. One principal investigator (WSB) is a board-certified, fellowship-trained orthopaedic foot and ankle surgeon who operated on all the patients and implanted all of the citrate-based bioceramic devices. The second principal investigator (LS) is a board-certified, fellowship-trained orthopaedic foot and ankle surgeon who practices at a different institution in a different state and had no involvement with the patients or surgeries that resulted in implantation of the devices. The initial radiographic assessments were performed in a blinded fashion using a checklist for each set of images.

Our primary study goal was to quantify bone tunnel enlargement associated with each citrate-based bioceramic device. To achieve this, a blinded separate quantitative assessment of bone tunnel enlargement on MRI was performed by the two principal investigators. Enlargement was quantified by measuring the width of any gap between the inner surface of the bone tunnel and the outer diameter of the device threads. It should be noted that a portion of each implanted device has no direct bony contact because a tendon graft is interposed between the outer surface of the device and the inner surface of the bone tunnel. This flattened, interposed tendon graft sits between the device threads and the tunnel on one aspect of the device, comprising approximately one-third to one-half of the device’s circumference. Therefore, only the aspect of the device directly adjacent to the bone and opposite the interposed tendon graft was utilized for measurement purposes. For assessment of bone tunnel enlargement, the study was designed so that discrepancies in MRI measurements between the two raters would be resolved through quantitative averaging. However, the intraclass correlation coefficient (ICC) was 1.0 (95% confidence interval 1.0 to 1.0), indicating perfect agreement between the raters with regard to width of gap measured to the nearest millimeter.

Our secondary study goals were to measure adverse MRI findings (such as bone marrow edema or device breakage) associated with the devices and to quantify the degree of osseous integration on MRI. An additional secondary goal was to document clinical complications in this surgical cohort.

Bone marrow edema was quantitatively graded on MRI images in blinded fashion by the two principal investigators with respect to the circumference of the device using a worst-case approach (the single slice that showed the largest area of bone marrow edema was the one used to grade the device). The following interval scale (out of 4) was used: 0 = no peridevice bone marrow edema present, 1 = bone marrow edema surrounding up to 25% of the device circumference, 2 = bone marrow edema surrounding 26% to 50% of the device circumference, 3 = bone marrow edema surrounding 51% to 75% of the device circumference, and 4 = bone marrow edema surrounding 76% to 100% of the device circumference.

Percentage intervals assessed on the five-point scale for bone marrow edema demonstrated high interrater reliability (prevalence-adjusted, bias-adjusted kappa [PABAK] = 0.92). Discrepancies in interval scale for bone marrow edema were adjudicated during a consensus review meeting attended by all investigators, using nominal group technique for quantitative grading [11].

The presence or absence of all other device-related adverse findings was assessed qualitatively on MRI in blinded fashion by the two principal investigators. These findings included peridevice fluid collections, peridevice osteolysis, peridevice intraosseous or soft tissue cysts, sinus tracts, and device breakage. Assessment for the presence or absence of these device-related adverse findings demonstrated high interrater reliability (PABAK = 0.92 for fluid and 1.0 for all others). For these measures, disputes were resolved in favor of the presence of the finding in order to capture the worst-case scenario.

MRI was also used to assess the degree of osseous integration between the device and surrounding bone. Osseous integration was defined as the lack of any distinct border between the outer diameter (threads) of the device and the surrounding cancellous bone on the aspect of the device opposite the tendon. Integration was quantitatively graded in blinded fashion by the two principal investigators with respect to the full length of the device according to the following interval scale (out of 4): 0 = no osseous integration along the device length, 1 = osseous integration up to 25% of the device length, 2 = osseous integration along 26% to 50% of the device length, 3 = osseous integration along 51% to 75% of the device length, and 4 = osseous integration along 76% to 100% of the device length. Percentage intervals assessed on the five-point scale for osseous integration demonstrated high interrater reliability (PABAK = 0.92). Discrepancies in interval scale for osseous integration were adjudicated during a consensus review meeting attended by all investigators using nominal group technique for quantitative grading [11].

### Clinical Assessment

We completed a retrospective evaluation of all patients’ charts to tabulate the presence of all clinical complications in our surgical cohort (including those that were potentially device related).

### Ethical Approval

This retrospective study was approved by the WCG Institutional Review Board (IRB tracking number: 20260197). Because of the retrospective nature of the study, the requirement for informed consent to include individual patient data was waived by the board, and patient data were anonymized to ensure confidentiality.

### Statistical Analysis and Sample Size Calculation

An a priori power analysis for bivariate correlation of our primary MRI parameter was based on data from a previously published study that measured bone tunnel enlargement [19]. We determined that to detect a 41% increase in tunnel diameter, with a power of 0.8 and a significance level of 0.05, a minimum number of seven devices would be necessary to avoid an insufficiently powered study.

Data points recorded during the initial assessment by the two principal investigators were analyzed for interobserver reliability using two-way mixed, single-measurement, absolute-agreement ICC for bone tunnel enlargement and PABAK for all other parameters.

## Results

### Bone Tunnel Enlargement

None of the 25 devices in 13 patients who underwent MRI analysis exhibited bone tunnel enlargement (Table 3). The gap on STIR sequences measured 0 mm for all 25 devices (Fig. 3). There was also no gap evident on fat-suppressed images (Fig. 4).

**Table 3.** MRI data

Patient	Duration of time between surgery and MRI in months	Specific implant and osseous location	Distance between outer circumference of implant and inner circumference of bone tunnel in mm	Grade of bone marrow edema (out of 4)^a^	Presence of other adverse MRI findings^b^	Grade of osseous integration (out of 4)^c^
1	48	Calcaneus	0	0	None	4
2	No MRI	Calcaneus				
3	No MRI	Calcaneus				
4	44	Lateral malleolus	0	0	None	4
		Talus	0	0	None	4
		Calcaneus	0	0	None	4
5	43	Lateral malleolus	0	0	None	4
		Talus	0	0	None	4
		Calcaneus	0	0	None	4
6	42	Lateral malleolus	0	0	None	4
		Talus	0	0	None	4
		Calcaneus	0	0	None	4
7	39	Calcaneus	0	0	None	4
8	39	Lateral malleolus	0	0	None	4
		Lateral talus	0	0	Fluid	2
		Anterior calcaneus	0	0	None	4
		Posterior calcaneus	0	0	None	4
		Medial malleolus	0	1	None	4
		Medial talus	0	0	None	3
9	36	Calcaneus	0	0	None	4
10	35	Calcaneus	0	Metal artifact	None	4
11	34	Calcaneus	0	0	None	4
12	34	Calcaneus	0	0	None	4
13	31	Talus	0	1	None	2
		Calcaneus	0	0	None	2
14	30	Cuboid	0	0	None	4
15	27	Calcaneus	0	0	None	4

a Grading scheme for bone marrow edema (out of 4): 0 = no peridevice bone marrow edema present, 1 = bone marrow edema surrounding up to 25% of the device circumference, 2 = bone marrow edema surrounding 26% to 50% of the device circumference, 3 = bone marrow edema surrounding 51% to 75% of the device circumference, 4 = bone marrow edema surrounding 76% to 100% of the device circumference.

b Each MRI specifically examined for other adverse periimplant findings, including fluid collections, osteolysis, intraosseous or soft tissue cysts, sinus tracts, and implant breakage.

c Grading scheme for osseous integration (out of 4): 0 = no osseous integration along the device length, 1 = osseous integration up to 25% of the device length, 2 = osseous integration along 26% to 50% of the device length, 3 = osseous integration along 51% to 75% of the device length, 4 = osseous integration along 76% to 100% of the device length.

**Fig. 3:**
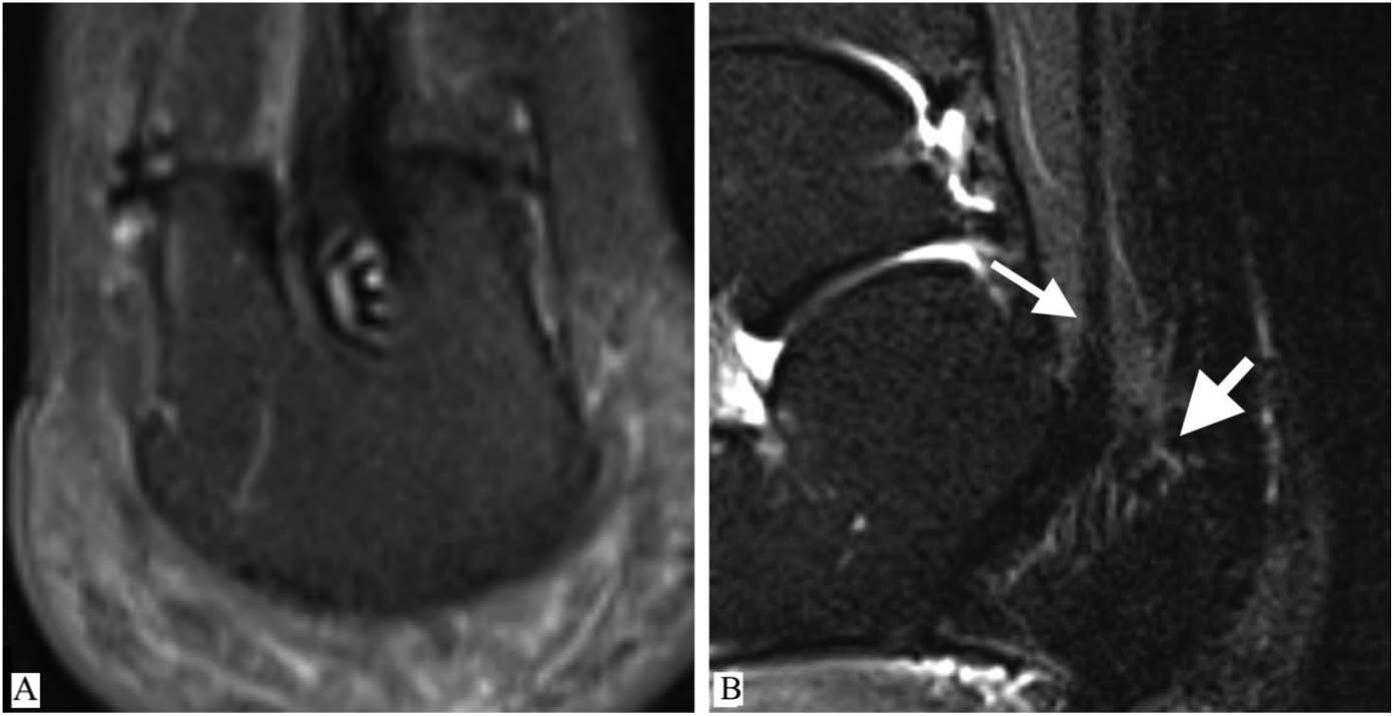
An MRI performed 3 years after FHL transfer shows no bone tunnel enlargement, with thread tips in contact with bone on the (**A**) coronal fat-suppressed image and (**B**) sagittal STIR image. The tendon (small arrow) is anterior to the citrate-based device (large arrow).

**Fig. 4:**
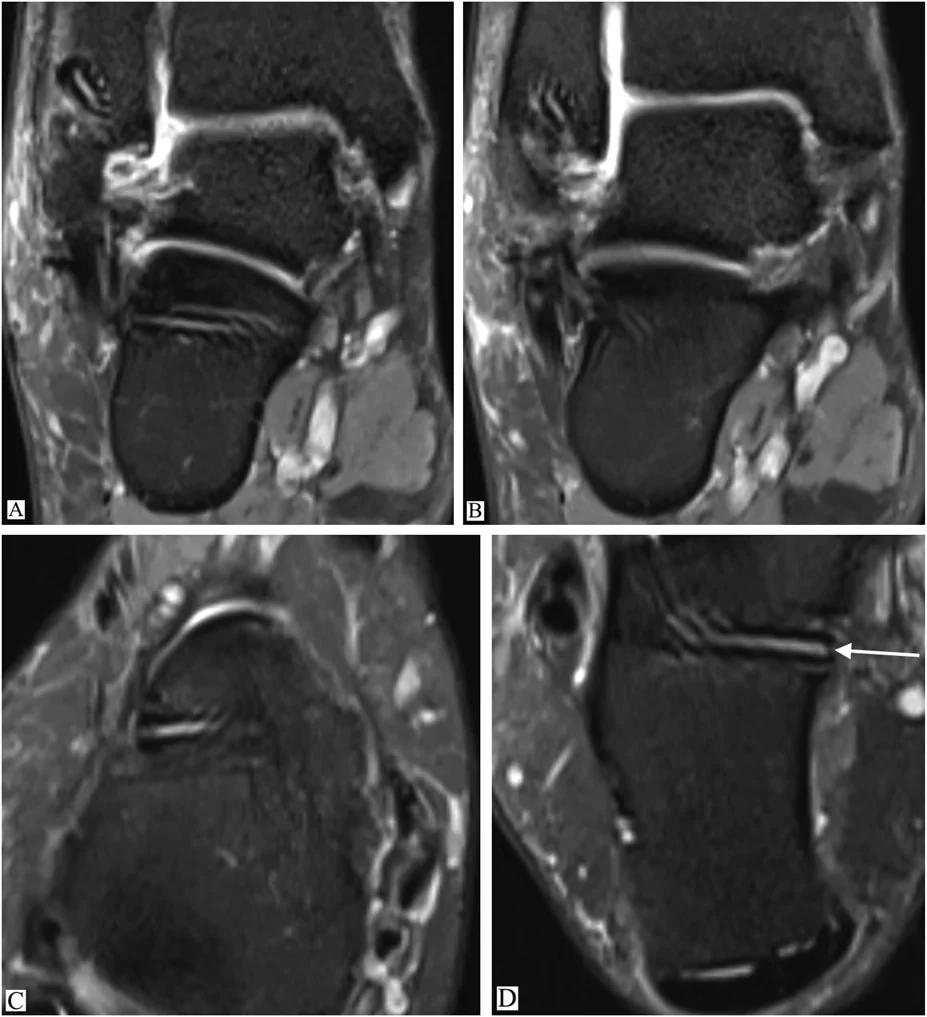
An MRI performed 3 years 6 months after ankle ligament reconstruction shows no bone tunnel enlargement around devices in the (**A**) calcaneus and lateral malleolus, (**B**) lateral malleolus, (**C**) talus, and (**D**) calcaneus in various T2-weighted fat-suppressed planes. The channel at the center of the device (arrow) fits the tip of the inserter.

### Bone Marrow Edema and Other Device-related Adverse Findings

This study showed very few signs of peridevice inflammation on MRI (Table 3). The median grade of bone marrow edema was 0 of 4 (n = 24). Twenty-two of 24 devices showed no sign of bone marrow edema, with a grade of 0 of 4. Two of 24 devices showed grade 1 of 4 surrounding bone marrow edema, corresponding to edema around 25% or less of the device circumference (Fig. 5). One device could not be accurately graded for bone marrow edema because of an adjacent metal screw that caused susceptibility artifact. None of the 25 devices showed the presence of peridevice osteolysis, intraosseous cysts, soft tissue cysts, sinus tracts, or device breakage. One of 25 devices showed fluid present at the surface abutting the tendon graft, but none at the bone-device interface.

**Fig. 5:**
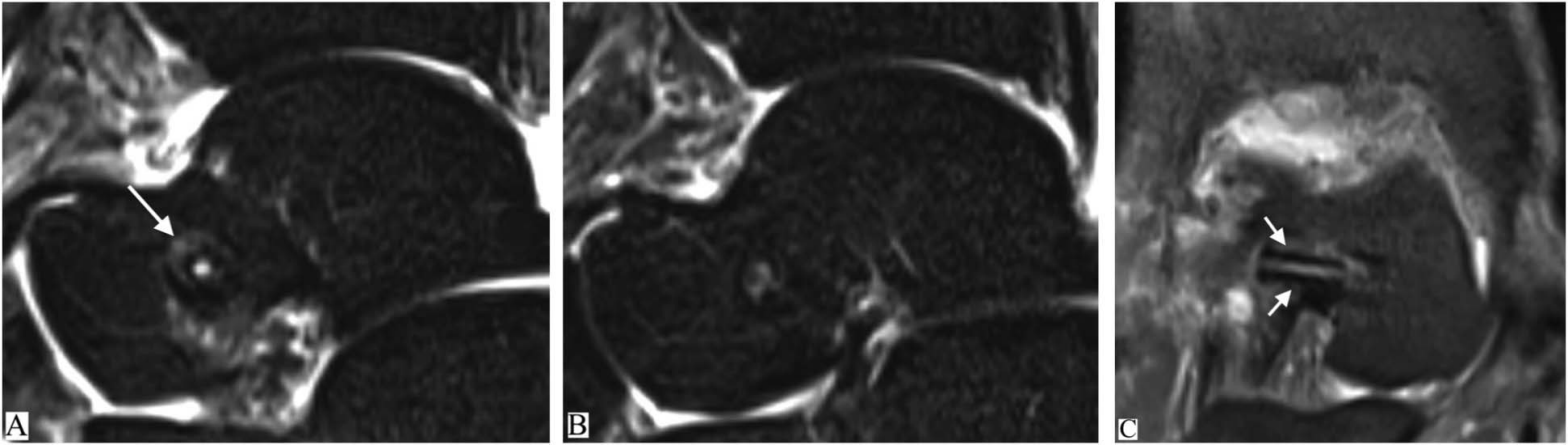
(**A**) Two years 7 months after this citrate-based device was inserted into the talus, bone marrow edema (arrow) was noted based on a single MRI slice using worst-case scenario methodology. (**B**) The adjacent slice on the same series shows no sign of bone marrow edema. (**C**) Despite the edema, the device still appears to be integrating with the bone on its dorsal and plantar surfaces (arrows).

### Osseous Integration

All 25 devices demonstrated some degree of osseous integration, and most exhibited near complete osseous integration along the bone-device interface (Table 3). The median grade of osseous integration was 4 of 4 (n = 25). Twenty-one of 25 devices were graded as 4 of 4, corresponding to integration of 76% to 100% of their length along the bone-device interface (Fig. 6). One device was graded as 3 of 4 (51% to 75% integrated) and three devices were graded as 2 of 4 (26% to 50% integrated). It was noted that in devices implanted for longer periods of time, the shape of the device changed as remodeling and osseous integration progressed (Fig. 7).

**Fig. 6:**
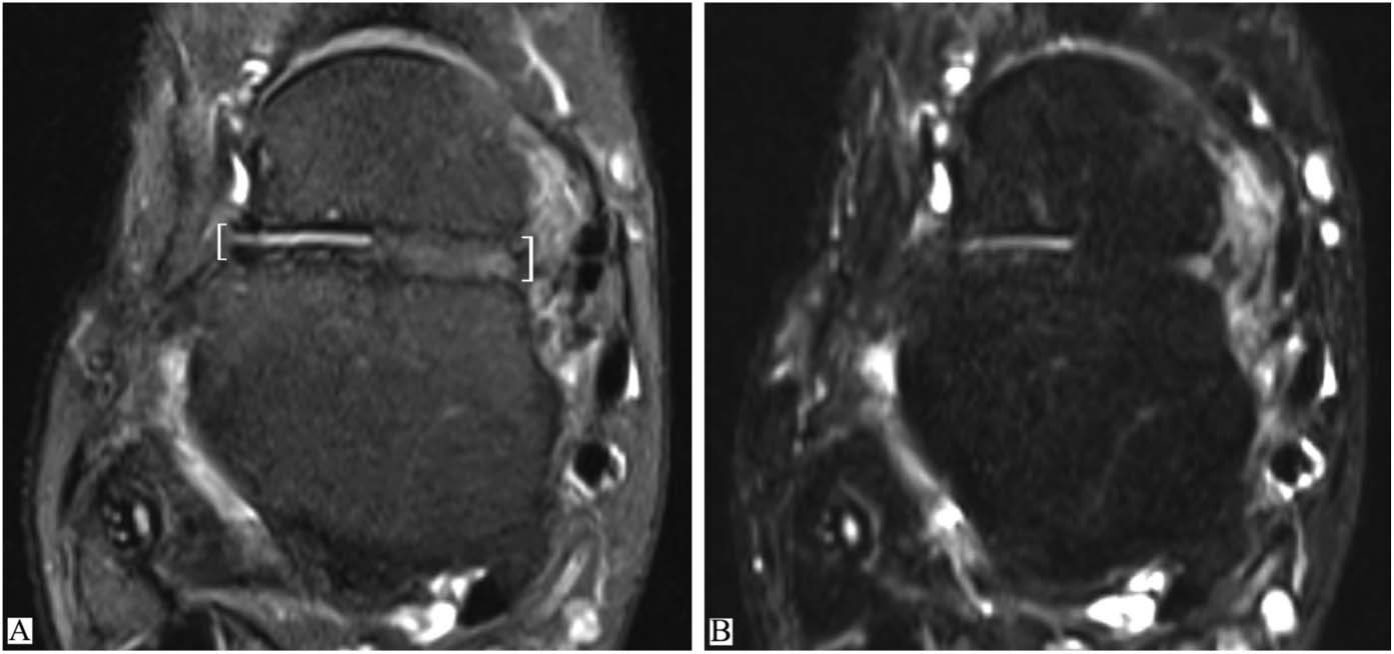
(**A**) Three years 8 months after implantation, tidemarks medial to the device on this T2-weighted fat-suppressed image show the original width of the bone tunnel in the talar neck (right bracket). In the lateral aspect of the talar neck, the width of the tunnel directly around the citrate-based device has narrowed as the surrounding bone has grown in around it (left bracket). The channel in the device used to accommodate the tip of the inserter is visible. (**B**) The identical MRI slice using STIR confirms that the device has integrated with bone, and the entire tunnel medial to the device has filled with bone.

**Fig. 7:**
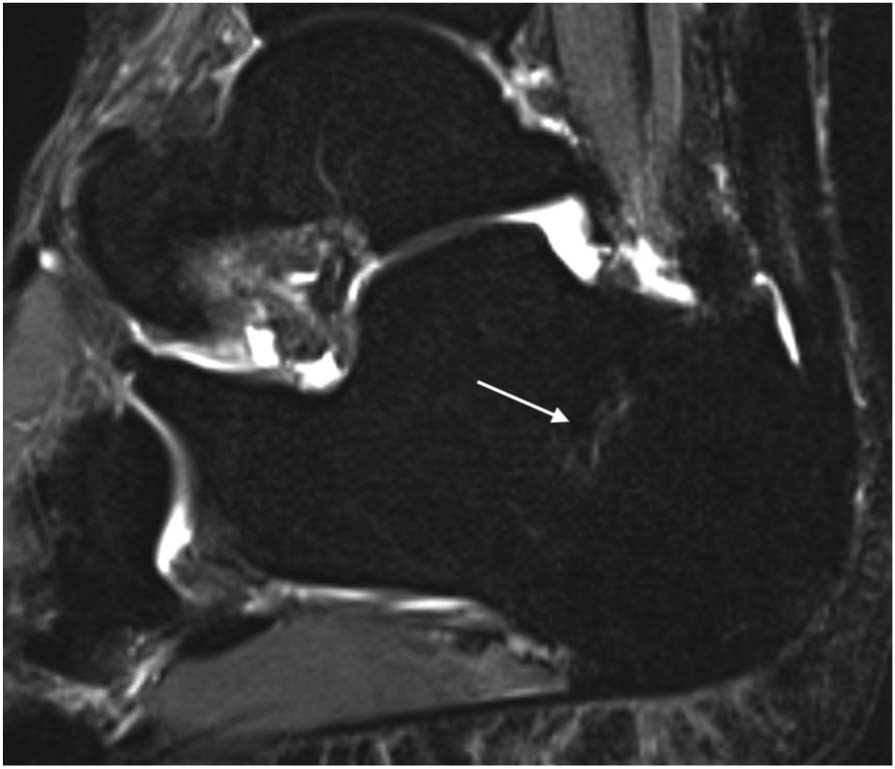
Four years after FHL tendon transfer, the threads on this citrate-based device are no longer visible, as the remaining hydroxyapatite component (arrow) undergoes osseous integration.

### Complications

Clinically, there were no device-related complications at a median follow-up of 36 months in the 13 patients who were accounted for at a minimum of 2 years (range 2 to 4 years). Ten of 13 patients did not develop any postoperative clinical complications. Of the three patients who did have complications, two were subcutaneous absorbable suture abscesses. Both resolved promptly after debridement and excision of the underlying suture material. The third patient had initially presented to the office with an open wound over his Achilles tendon and a 14-month history of chronic osteomyelitis after fixation of a calcaneal tuberosity fracture. This patient underwent multiple debridements and courses of intravenous antibiotics and was deemed free of infection after two successive surgeries (without antibiotics), which showed negative intraoperative cultures. Ultimately, the patient underwent Achilles tendon reconstruction using an FHL tendon transfer with a citrate-based device but developed recurrent wound drainage postoperatively. After one further debridement and course of antibiotics (a decision was made to retain the citrate-based device), the patient returned to work as a plumber’s assistant and remains clinically and radiographically free of infection 41 months after the final debridement.

## Discussion

Resorbable fixation devices offer potential advantages over permanent devices but have gained limited acceptance in orthopaedic surgery because of osteolytic and inflammatory effects. Citregen, a resorbable material made from 40% poly(octa-methylene-citrate) and 60% hydroxyapatite, has recently become available in the United States. Our study used MRI to illustrate two properties of a citrate-based bioceramic device that distinguish it from those that are lactate based or glycolate based and that have the potential to improve patients’ clinical results in perceptible ways. First, unlike thermoplastic polymers, the citrate-based material in this study did not lead to substantial peridevice inflammation. Rather than being an innocuous imaging phenomenon, peridevice inflammation has real clinical consequences [15, 16, 18]. Second, in this study, none of the citrate-based devices was associated with bone tunnel enlargement, and most demonstrated near-complete osseous integration, which contrasts with findings from previous analyses of thermoplastic polymers showing the opposite effect, with tunnel widening and osteolysis [1, 3, 7, 12, 16, 19, 21, 24]. Although these findings are promising, this was a small series. Therefore, the results presented here should not serve as the basis for widespread adoption of this new material until or unless other rigorous, independent studies replicate our observations.

### Limitations

This study had several important limitations. First, one of the primary investigators (WSB) is both an investor and consultant for the company that produces these devices. This investigator was one of two who rated the MRI findings. Involvement of this investigator in reading MRIs was admittedly problematic because of bias in favor of device performance. However, this investigator was the operating surgeon with the most experience with citrate-based bioceramic devices, so we felt that he had a unique perspective that would be helpful when using nominal group technique to devise a quantitative grading system for marrow edema and osseous integration. We hope that this limitation may have been partially ameliorated by the blinded participation of another experienced investigator (LS) who has no history with the company, has never implanted a citrate-based device, and independently reviewed all MRIs. A second safeguard against investigator bias lies in our conservative MRI adjudication methods, which for device-related adverse findings were always resolved in favor of the presence of the finding, or the worst-case scenario. Worst-case scenario was applied to disputes between investigators and also when adverse findings were noted on only a single MRI slice (Fig. 5).

Second, the study had a very small sample size. Adequate sample size matters because it allows us to detect problems that may be important but occur less frequently. With a larger sample, it is possible that device-related complications or adverse MRI findings would have become apparent. This limitation is compounded by the fact that several of the 13 patients had multiple devices implanted in the same extremity. Because it is expected that a host response to a particular material may remain relatively constant among devices, analyzing more than one device in a single patient dilutes the study findings. Thus, we caution the reader that the sample size here should be considered 13 (the number of patients with complete follow-up) rather than 25 (the number of implanted devices) because multiple observations in the same patient should not be considered biologically independent. In spite of this limitation, we believe that our MRI findings can still be trusted. Previous imaging studies of resorbable devices have demonstrated that periimplant osseous findings are often not subtle. The effect sizes in those studies are dramatically large, with expansion of bone tunnel volume of > 40% [3, 19, 24]. Our sample, although small, consistently showed no bone tunnel enlargement, and the relative absence of inflammatory findings was also uniform. Such high consistency between patients, coupled with the large effect size noted with alternate resorbable devices, lends validity to our study.

A third limitation concerns the lack of a matched control group. It would have been ideal to include a sample of patients who had undergone soft tissue fixation with lactate-based thermoplastic devices for purposes of direct comparison. Although readers may be tempted to contrast our data with historical MRI findings obtained with the use of alternate resorbable devices, we suggest instead waiting for head-to-head studies before drawing any conclusions.

Fourth, our study was retrospective, and thus prone to assessment bias, transfer bias, and selection bias. The most likely in our series was assessment bias, which concerns the quality of the information that we collected. Our clinical information would have been far more detailed and reliable if we had administered a baseline patient outcome instrument before surgery and then readministered it on a periodic basis. The reader may accept the fact that in our very small cohort, we saw no device-related complications but should remember that we did not look at actual endpoints from the patient’s perspective in a validated fashion. An element of transfer bias also existed as two of 15 patients did not undergo MRI. Patients who are lost to follow-up may have less favorable outcomes, and this can skew results to look more positive than they were. However, our patient follow-up of 87% exceeds the 80% figure that is often considered a sufficient threshold in this type of study. With regard to selection bias, this was minimal in our series. Because all patients undergoing tendon transfer or ligament reconstruction during the period of time in question received this citrate-based bioceramic device, our sample adequately represented our study population.

A fifth limitation was the lack of a predefined, validated methodology for assessing bioresorbable devices in the foot and ankle. The only published articles we could locate on this subject focused on the effects of bioresorbable pins, which are much smaller than the devices we used, and thus not evaluated in the same way [7, 15, 16]. We feel that our grading system, which uses the distance between the inner circumference of the bone tunnel and the outer circumference of the device as a measure of bone tunnel enlargement, is superior to other published approaches concerning ACL reconstruction. These alternate methods often rely on comparison between the size of a utilized reamer and digital measurement of tunnel diameter. This method is imprecise because of spatial distortion from nonlinearity of gradient fields that can result in discrepancies of several millimeters between actual measurements and MRI measurements. However, our methodology is also untested, and in the event of graft hypertrophy within the tunnel that displaces the interference device toward the opposite wall, it might minimize any measurable gap on that side. In addition, because we based our power analysis on a previous paper that utilized an alternate methodology, this might have led to our study being underpowered. We also recognize that our grading system for bone marrow edema, by focusing on spatial extension and not on signal intensity, may underrepresent biological severity.

As a sixth limitation, the mean follow-up time in this study of 37 months, with a minimum of 27 months, was early. However, in most patients, it did exceed the anticipated approximate 3-year degradation period for the polymer component of the devices. Accordingly, minimum follow-up of 36 months is suggested for future studies involving citrate-based devices with similar size, shape, and composition. Finally, a subanalysis by sex was not performed in this study. Our cohort was simply too small, with only five females, to stratify these data in any meaningful way. Therefore, the reader should not assume that findings drawn from a mixed sample of males and females would apply equally to both sexes when considered separately.

### Bone Tunnel Enlargement

Thermoplastic devices consisting of lactic and glycolic acid are commonly associated with osteolysis and bone tunnel enlargement [1, 3, 7, 12, 16, 19, 21, 24], but our analysis of this citrate-based bioceramic device found no bone tunnel enlargement at a minimum follow-up of 2 years after surgery. One proposed etiology of osteolysis and tunnel enlargement is acid dumping from bulk degradation of polymer, which precipitously lowers the pH of surrounding bone and soft tissue. Device manufacturers over the past 10 years have endeavored to minimize the effects of acid dumping by incorporating a bioceramic (such as hydroxyapatite or tricalcium phosphate) into the polymer to act as a buffer. In conjunction with other chemical adjustments to slow polymeric degradation, this modification has served to drastically decrease the likelihood of sterile abscesses [1], draining sinus tracts [14], and other severe short-term complications [6] that were common in the earlier days of thermoplastic devices. The tradeoff is that by slowing the resorption rate, sometimes to more than a decade, the surrounding bone is exposed to the acidic by-products of degradation for a much longer duration of time. This has manifested itself in a more insidious fashion, with bone tunnel enlargement [3, 12, 19, 24], peridevice inflammation [15, 16, 18], and long-term lack of osseous integration [4, 20, 21, 23]. Some studies utilizing advanced imaging of lactate-based interference devices have demonstrated an increase in bone tunnel volume of > 40% [19, 24], whereas in our study, both raters found that the bone tunnel consistently remained in direct contact with the outer diameter of the citrate-based bioceramic device along the bone-device interface. To be clear, this was a very small case series consisting of only 13 patients, and we are not using the limited data presented here as a recommendation for the adoption of this material. Instead, this study merely reports on early MRI findings in an initial group of patients after implantation. The data outlined in this article may serve as a future starting point for further investigation that will confirm or refute our findings.

### Bone Marrow Edema and Other Device-related Adverse Findings

In our study, two of 25 citrate-based bioceramic devices showed signs of peridevice bone marrow edema, which was mild and confined to < 25% of device circumference. In contrast, the evidence reports that bone marrow edema occurs in as many as 67% of patients treated with lactate-based thermoplastic devices in the talus [15]. Other adverse findings were similarly uncommon, with one of 25 devices showing fluid adjacent to the tendon graft. Reasons for the scarcity of an inflammatory reaction with citrate-based bioceramic devices are speculative, but based on bench research and animal models, this effect may be attributed to four issues. First, the completely amorphous and crosslinked structure of thermoset polymers does not permit bulk degradation, thus preventing a late-stage acid dump [27]. Second, the citrate-based bioceramic composite contains 60% hydroxyapatite, which matches native bone content, providing a biomimetic composition [17, 25]. By comparison, thermoplastic polymers become too brittle for insertion when the bioceramic content exceeds 35% [22]. Third, citrate is highly conserved in bone (85% of citrate in the human body resides in the skeleton), and when released, promotes osteogenesis by improving local metabolism through the citric acid cycle [5, 9, 26]. Fourth, the modulus of elasticity of the citrate-based bioceramic composite (mean ± SD 314.3 ± 53.9 mPa) [17] was comparable to that of trabecular bone in the foot and ankle (10 to 3000 mPa) [13], minimizing bone resorption and mechanical wear debris [22].

### Osseous Integration

Osseous integration of citrate-based devices in our study was high, with all 25 devices showing some degree of integration and 21 of 25 devices showing near-complete integration along the bone-device interface. A number of studies have endeavored to discover the extent of osseous integration around thermoplastic devices by using advanced imaging, with variable results. One randomized trial evaluated 40 patients who had undergone ACL reconstruction fixed either with screws made of either titanium or poly-L-lactic acid with hydroxyapatite (PLLA-HA). Patients underwent MRI at 2, 5, and 13 years, and they had CT scans at 13 years as well. Although the presence of hydroxyapatite may buffer the acidification of surrounding tissue by PLLA to some degree, the authors noted that even at 13 years, “tunnel volumes remained largely unchanged, with minimum ossification” [20]. These findings are consistent with those reported in a previous cohort study on the use of poly(lactic-co-glycolic acid) screws in ACL reconstruction, which concluded that “although these newer generation screws were designed to promote osseointegration, no tunnel narrowing was noted, and in most patients, the remains of the screws were present at approximately 3 years” [4].

### Complications

The frequency of implant-related complications with the use of thermoplastic devices may approximate 10% [8]. We noted three complications in 15 patients at short term, none of which was implant related, in our very small series of patients treated with citrate-based devices. Although our study was not large enough to draw firm conclusions about risks and harms, the lack of a clinically apparent inflammatory reaction in any of our patients aligns well with our MRI findings. A much larger series of patients assessed with longitudinally administered outcome measures would be necessary to determine the true frequency of clinical complications.

### Conclusion

In our study, common problems associated with other resorbable devices were not seen. We noted no bone tunnel enlargement and excellent osseous integration. Although the findings are promising, our series was very small and had multiple limitations. This resorbable biomaterial is composed of a citrate-based thermoset polymer with hydroxyapatite and has been approved by the FDA for musculoskeletal repair, but our study should not serve as a basis to recommend widespread use of this material without publication of larger and more rigorous studies that independently replicate our findings. Future research should seek to confirm or refute these results through clinical trials that prospectively compare citrate-based devices with those incorporating thermoplastic polymers.
